# Current Trends in Food Processing By-Products as Sources of High Value-Added Compounds in Food Fortification

**DOI:** 10.3390/foods13172658

**Published:** 2024-08-23

**Authors:** Helen Stephanie Ofei Darko, Lama Ismaiel, Benedetta Fanesi, Deborah Pacetti, Paolo Lucci

**Affiliations:** Department of Agricultural, Food and Environmental Sciences, Università Politecnica delle Marche, 60131 Ancona, Italy; helenstephanieofei.darko@unito.it (H.S.O.D.); l.ismaiel@univpm.it (L.I.); d.pacetti@univpm.it (D.P.); p.lucci@univpm.it (P.L.)

**Keywords:** animal, fish, vegetables, fruit, waste, loss, antioxidants, phenolics, functional foods

## Abstract

Along the food production chain of animal, fish, and vegetable products, a huge amount of by-products are generated every year. Major nutritional, financial, and environmental advantages can be achieved by transforming them into functional ingredients for food formulation and fortification. In this review, we investigated various conventional and emerging treatments recently employed to obtain functional ingredients rich in proteins, fibers, and bioactive compounds from vegetables, fish, meat, and dairy by-products. The optimal enrichment level in food as well as the nutritional, techno-functional, and sensory properties of the final food were also discussed. Novel technologies such as ultrasounds, microwaves, and high pressure have been successfully adopted to enhance the extraction of target compounds. The functional ingredients, added both in liquid or powder form, were able to improve the nutritional quality and antioxidant potential of food, although high levels of fortification may cause undesired changes in texture and flavor. This review provides important considerations for further industrial scale-up.

## 1. Introduction

One component of the Green Deal that concentrates on the food system is the Farm to Fork approach, which employs the circular economy model to increase the sustainability of food production. Improving the utilization of food waste, surplus, and by-products within the framework of food supply chains would be necessary to close the loops created [1]. Ultimately, three-quarters of edible food meant for human consumption is lost or wasted annually worldwide within the production and supply chain, amounting to around 1.3 billion tons [2]. These losses result from the entire food value chain, which starts with harvesting and continues through postharvest, industrial, and commercial processing before ending with consumer consumption. Additionally, by-products of food processing that are side streams obtained after target products are produced throughout all food production sectors also count as food losses [3]. Food processes such as vegetable oil extraction, starch, juice, and sugar production and fish and meat processing, resulting in animal waste side streams such as bones, offal, and hides, and whey protein from cheese processing generate waste or by-products at extremely intriguing volumes. A large portion of these products is typically thrown out during processing or traditionally used as compost or animal feed [4]. More recently, however, there has been investigation into their potential as alternate energy sources [5].

Currently, utilizing by-products for food fortification has emerged as a substitute strategy. Specifically, when food processing by-products contain significant amounts of favorable and valuable compounds like proteins, vitamins, minerals, fiber, and essential fatty acids, they can be applied in the food and pharmaceutical industry, thereby increasing their potential to be included in the human diet [5,6]. In addition, it is anticipated that the human population will grow by 2050, highlighting the need to create a sufficient food supply to ensure that food production will be possible in the future [7]. Efficient by-product usage has garnered significant attention in recent times due to its potential to improve sustainability, reduce waste disposal, and generate value-added goods for the food sector [8]. By-products from fruits and vegetables, for instance, are known to have a significant quantity of dietary fiber and bioactive components with significant biologically active components like phenolic and antioxidant compounds [6]. Likewise, the by-products of meat and dairy processing can provide valuable substances such as proteins, lipids, chitin, collagen, and whey proteins that can be used for food fortification [9,10]. While there has been previous discussion on the use of fish by-products in food fortification, there has not been much recent discussion about the relationship between fish by-products and new food processing techniques. This review not only emphasizes how crucial it is to pretreat fish by-products, but it also offers recommendations for the best way to use these substances in food formulation.

**Figure 1:**
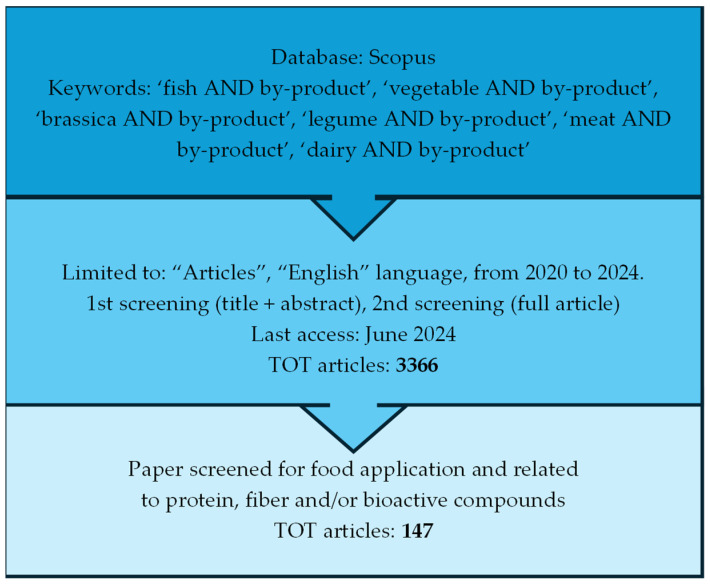
Flow chart of the methodology applied for screening of articles.

Many biochemical, chemical, physical, or mechanical techniques have been used to extract bioactive molecules, provided that the procedures are selective, microbiologically safe, and do not change or modify the compounds. The extraction field is expanding rapidly these days, and as a result, there is a lot of interest in finding ways to expedite the extraction process, boost quality, and protect bioactive compounds [11]. This topic is novel given that there is a growing body of research and interest in the innovative extraction techniques that can be used to successfully recover and stabilize bioactive compounds with improved functional characteristics from by-products of the food industry as well as food applications that successfully integrate high-value compounds into food systems. As a result, a more sustainable and environmentally friendly food system can be developed. In this review, we aimed to provide an overview of the most recent developments and/or technologies in the valorization of food processing by-products from vegetables, meat, fish, and dairy value chains, with a focus on the production of protein-, fiber-, and bioactive-rich functional ingredients and their incorporation into food. Comprehensive terms (‘fish AND by-product’ (1075), ‘vegetable AND by-product’ (703), brassica AND by-product’ (117), ‘legume AND by-product’ (72), meat AND by-product’ (790), and ‘dairy AND by-product’ (606)) were used to obtain a broad selection of articles published in the period from 2020 to 2024 from the Scopus database. Consequently, a total number of 147 articles were considered for full screening and discussion (Figure 1).

## 2. Characterization of Food Processing By-Products Based on Their Nutritional Benefit, Treatment, and Food Application

### 2.1. Protein

The demand for protein greatly increased both to meet consumers’ needs for healthy diets and to face undernourishment in some poor countries. Moreover, life cycle assessment of meals derived from animals and plant-based, high-protein alternatives has been demonstrated to broaden the supply of sustainable high-protein food options, making these products more economically attractive [12]. Here, the importance of extracting proteins from alternative sources such as vegetable and animal by-products is discussed.

Leaves, stalks, press cakes, spent grains, and aquafaba are among the most common fruit and vegetable by-products obtained during food processing. These by-products represent an alternative source of proteins. Therefore, they can be valorized by transformation into food ingredients either in liquid form or powder. The extraction of vegetable oil from seeds, nuts, or fruits, usually obtained through screw-pressing or employing solvents, provides a large amount of residues, also called press-cake or meal, that can be ground and used as a protein-rich ingredient in quantities of ~30–40% [13,14,15]. The removal of lipid fraction from by-products can also be a pre-treatment for further purification processes to obtain a high protein concentration or improved functional properties. The conventional process is the wet method, consisting of alkaline extraction followed by isoelectric precipitation. Basically, ground meal is dispersed into water with a sample-to-solvent ratio of about 1:10–1:15. Later, sodium hydroxide (NaOH) is added to achieve an alkaline pH, i.e., between 9 and 12, which allows to solubilize/extract proteins while removing insoluble fibers [16]. Mild treatments (pH 9–10) have been adopted for rapeseed cake and quinoa okara to obtain stable emulsions with low particle size [17,18]. Harsh treatments (pH > 10) may lead to protein denaturation, therefore reducing recovery yield. In some cases, a stronger alkalinization (pH 12) was necessary to increase protein and polysaccharide solubilization, such as for spent brewer’s yeast, which is characterized by strong and thick cell walls, and to improve emulsion stability [19]. The time–temperature relationship of alkaline treatments may vary from 30 min at 55 °C to 2–4 h at 20–25 °C [17,19]. Generally, temperatures above 60 °C may induce protein denaturation and gelation, reducing the protein concentration of the final ingredient. The time should be adequate to obtain protein dispersion without achieving protein saturation level. Afterward, protein precipitation is induced by adding hydrochloric acid (HCl) to adjust the pH towards the isoelectric point, which is usually between pH 3 and 5 for plant proteins [17]. Besides this, innovative non-thermal technologies such as ultrasound (US) and fermentation have been investigated to increase the protein content of the final food ingredient. Cavitation phenomena generated by US induce protein denaturation favoring solubilization [18,20]. Kasapoglu et al. applied US homogenization (405 W for 2.5 min) and obtained a coconut oil by-product-based drink with a protein content double the amount found in other commercial beverages [20]. On the other hand, a high-intensity US treatment (75% pulse amplitude for 10 min) applied to alkalized okara solution allowed it to have a protein concentration four times higher than the untreated control solution, demonstrating that the application of this green technology may induce conformational changes in the protein structure favoring hydrophobic interactions among proteins, which tend to aggregate and precipitate during the resting phase [18]. Additionally, fermentation was recently exploited by Castellanos-Fuentes et al. as a strategy to increase protein and fiber content while decreasing sugars as well as to improve the physicochemical and functional properties of the soy meal-based food ingredient [13]. Valorization strategies based on fermentation of vegetable by-products were deeply discussed by [21].

As an alternative to wet methods, dry fractionation allows to produce protein-rich ingredients separating the fine (protein-rich) and the coarse (starch-rich) fractions without water and chemical consumption and maintaining proteins in their native state. On the downside, the final ingredient is characterized by the presence of antinutrients and low purity. For example, Squeo et al. employed this technique with defatted durum wheat cake using an air classifier to increase the protein yield, yet the protein content did not exceed 31% [22]. Therefore, the advantages and disadvantages of introducing an additional protein concentration step should be carefully evaluated along the production process of the food ingredient.

To this end, many authors did not perform any protein concentration but employed the whole by-product flour as a protein source food ingredient. As for Brassica species [23,24,25] and black gram [26], by-products were stabilized (e.g., blanching and drying), milled, and sieved, with a protein concentration obtained ranging from 23 to 31%.

Emulsions, pasta, bakery goods, snacks, beverages, ice creams, and burgers are among the various food applications that have been recently investigated. Different by-product sources such as artichokes bracts, tomato seeds and skin, and broccoli leaves have been used to enrich pasta (up to 30% of enrichment). In some cases, it was possible to improve the protein content and the amino acid composition of pasta. However, the higher the enrichment level, the higher the cooking loss, an important parameter that should not exceed 10% to have an acceptable product [23,27,28]. Soy- and broccoli-based ingredients (53% and 29% protein, respectively), when used in a concentration of 5–6%, were able to improve the protein and mineral content of gluten-free bread as well as color, specific volume, and bake loss [13,24]. For muffins from cauliflower by-products [25] and protein bars from durum wheat cake [22], the optimal enrichment level was assessed at 10% both in terms of nutritional quality (higher protein and fiber content) and physicochemical parameters (e.g., color and hardness). It was possible to almost triple the enrichment level using nut by-products while maintaining good acceptability, as was tested for cashew kernel, soy okara, and peanut meal in the formulation of vegan burgers and snacks, respectively [14,29,30]. Protein-rich ingredients obtained from different by-products such as rapeseed cake [17], quinoa okara [18], aquafaba [31], and spent brewer’s yeast [19] were investigated to develop emulsions. The concentrations varied from 0.1 to 6% when the food ingredient was added in the powder form and from 20 to 35% for liquid. In general, the emulsions showed good stability and low particle size. Canned chickpea aquafaba was also found as a good egg white replacer for meringue production [32].

Fish by-products provide great sources of protein (>75%), making them suitable for preparing and fortifying functional food products. Lately, they are used to cover a wide range of food applications, such as fish sausage, fish balls, coating films, emulsions, umami compounds, and ready-to-eat paste. Protein from fish by-products is subjected to hydrolysis before utilization and various concentrations (0.1–27%) have been applied to fortify food products like yoghurt and pasta. Conventionally, there are two main methods for protein extraction and hydrolysis, namely alkaline and enzymatic treatments.

Alkaline treatment can effectively solubilize protein, and it has been applied to fresh, frozen, and fermented by-products [33,34]. Samples are usually soaked with different concentrations of NaOH for various time slots. For example, alkaline treatment with 3% NaOH for 1 h was suitable to increase the concentrations of calcium and phosphorus in fortified fish sausage [33]. On the other hand, enzymatic treatment is widely applied using different types of enzymes (i.e., alcalase, protamex, flavourzyme, neutrase, trypsin, and papain). The raw material is typically minced and mixed with water. The pH and temperature are then adjusted to incubation conditions. After incubation, enzymes are inactivated. The mixture is centrifuged to separate the liquid phase. This liquid is mainly dried using a spray dryer or a freeze dryer to obtain hydrolysates.

The incubation temperature for alcalase is usually near 60 °C, and the pH is 7.6–8 for 85–180 min. Under these conditions, the protein content was increased in fortified biscuits and soup with fish by-product hydrolysates [35,36]. Additionally, in deep-fried battered squid containing brown stripe red snapper, alcalase hydrolysate showed efficiency in the reduction in oil binding capacity and increase in crispness [37]. Alternatively, a hydrolysis reaction can be achieved using a combination of enzymes depending on the desired target, such as protamex and flavourzyme at 50–55 °C for 2.5 h to improve the quality of fish sauce [38], or a mix of protamex (pH 7.0, 50 °C), alcalase (pH 8.0, 55 °C), neutrase (pH 7.0, 45 °C), and flavourzyme (pH 6.6, 50 °C) in a snack designed for physically active people [39]. Zhang et al. reported that trypsin and alcalase hydrolysates had stronger ABTS scavenging activity than papain and neutrase hydrolysates, suggesting the former as potential natural antioxidants [40]. Fortified yoghurt with 0.15% of flavourzyme hydrolysates from parrotfish by-products had considerable values for color, texture, and overall acceptance values [41]. Papain hydrolysate from yellowfin tuna red was employed as a fortifying and stabilizing agent in a mayonnaise emulsions system, replacing 50% of egg yolk. The resulting emulsions expressed a high quality with low particle size and enhanced oxidative and physicochemical stability [42].

Besides conventional hydrolysate production, some authors reported the use of cheap, eco-friendly, low-energy consumption technologies to speed up the enzymatic hydrolysis of proteins. For instance, US was used as pretreatment before the addition of the protamex enzyme, improving the extraction yield (76.34%) of umami compounds [43]. Moreover, the combined impact of natural deep eutectic solvents (NADES) and US pretreatments was studied as an extraction medium at 190 °C for the subcritical water hydrolysis of shrimp waste to be used as a biodegradable film with a high amount of protein [44].

In addition, there is increased attention on emulsions and microencapsulation prepared and stabilized with fish by-products including oil, gelatin, protein extracts, or protein hydrolysates as ready-to-use products or further application such as surimi. Gelatin has been isolated and investigated as a potential emulsifying, foaming, and thickening agent. Extracting gelatin has been subjected to various methods, including hot water-pretreated gelatin, acetic acid-pretreated gelatin, sodium hydroxide-pretreated gelatin, and enzyme-pretreated gelatin [45]. The quality and gel strength might differ depending not only on the mechanisms of extraction but also on the type of fish. Yang et al. applied pH shifting using 0.1 M NaOH and 0.05 M acetic acid on different fish skin to extract gelatin and further use it for fish oil-loaded gelatin-stabilized emulsions [46]. Gelatins and emulsions of catfish and silver carp had higher gel strengths and lower creaming index values than marine fish skin gelatin [46]. Similarly, high-value gelatins with strong gel values and significant emulsifying capacity were obtained through pepsin enzyme pretreatment after the removal of non-collagenous protein, fat, and minerals. The authors also claimed it has potential as a foaming agent for ice cream and marshmallows [45].

Pink perch surimi by-products were employed to obtain protein hydrolysate for further microencapsulation. The protein hydrolysate conditions were optimized using response surface methodology (RSM). RSM was chosen as an excellent statistical method for determining the optimal process conditions by decreasing the number of experimental trials while covering the interactions between parameters and decreasing the time and cost of tests. The protein hydrolysate produced had the highest yield (17%), good functional properties, and moderate antioxidant activity using the alcalase enzyme under the following conditions (57.9 °C, 85.8 min, with 0.15% (*v*/*w*) enzyme to substrate ratio) [47]. Furthermore, the antioxidant activity of microencapsulated, optimized protein hydrolysates was preserved; their bitterness and fishy odor were significantly decreased; and their overall acceptability was increased [47]. Optimizing protein hydrolysis conditions of surimi processing by-products proved a great potential to fortify various food formulations and provide value-added products. Correspondingly, surimi protein hydrolysate was produced with enzymatic interaction using alcalase and trypsin. The produced hydrolysate was added to silver carp surimi to prevent protein oxidation and enhance the gel properties [48]. Direct extraction of protein by thermal coagulation gave better physicochemical properties than enzymatic protein hydrolysates when preparing emulsions [49]. In another study, a simple pH shifting using 2 mol/L NaOH and HCl was applied for salmon by-products to obtain lyophilized protein powder. Subsequently, the latter was mixed with sodium alginate to encapsulate probiotics to improve its viability under pasteurization and gastrointestinal conditions [50]. Likewise, the encapsulation efficiency of corn oil was enhanced when using *Sardinella aurita* protein isolate spray-dried solution (4%) combined with maltodextrin [51]. Catfish protein concentrate extracted from catfish by-products with 0.5 N NaHCO_3_ was used to enrich microencapsulated highly nutritional fish oil with >17% protein [52].

It is worth noting that protein content was improved in pasta enhanced with 10% seabass by-product concentrates, which were obtained simply by dipping in saline solution (8%) and drying in the oven at 60 °C for 24 h [53,54,55]. Moreover, high-protein cereal bars and nuggets based on fish by-products were washed, minced, and cooked at 60 °C and 180 °C, respectively [56,57]. Pressure-cooking was applied to Nile tilapia by-products followed by pressing and drying at 90 °C for 24 h to obtain fish flour with high protein content (55.41%) [58].

Meat by-products including bones, heart, kidney, liver, tongue, skin, horns, hoofs, blood, tendons, visceral organs, and gastrointestinal tract can be valorized as well considering their content of high-value protein [6,59,60]. Valorizing these by-products to produce protein for food applications is typically subjected to enzymatic hydrolysis.

As in fish by-products, flavourzyme, papain, bromelain, and alcalase are the most-used enzymes. Alcalase showed a higher degree of hydrolysis compared to other commercial ones due to its ability to break peptide bonds [61]. It was the most effective enzyme for the generation of antioxidant peptides, followed by neutrase, papain, and pepsin [62]. On the other hand, flavourzyme was the best alternative for the formation of flavor compounds [61]. The required incubation conditions for each enzyme must be respected. Da Silva Bambirra Alves et al. also used alcalase to prepare chicken blood meal hydrolysates at optimal conditions of 50 °C, pH 8.5, and enzyme/substrate (E/S) ratio of 6.5% [63]. Mattohti et al. used a mix of neutrase (100 U/mg, 45 °C, pH 7.0), alcalase (400 U/mg, 50 °C, pH 9.5), papain (1000 U/mg, 55 °C, pH 6.5), and Pepsin (300 U/mg, 37 °C, pH 2.8) in the preparation of hydrolysates with antioxidant characteristics from horse bone marrow [62].

Meat by-product pre-treatment is usually applied prior to hydrolysis. For example, de Souza Fontes et al. subjected goat viscera to a heat treatment at 90 °C for 15 min in a water bath to inactivate the endogenous enzymes to inhibit competition for the active site of the proteins during enzymatic hydrolysis [64]. Likewise, fat was removed from meat by-products either using solvents [62] or heated water [65]. This step enhances the accessibility of enzymes to the active site during the hydrolysis process and raises the protein content [66].

Recently, innovative technologies have emerged to produce high-value enzymatic hydrolysis products. For instance, high-voltage electrostatic field and US, alone or in combination, improved the extraction rate of protein, the degree of hydrolysis, and flavor sub-stances in meat and bone residue [67]. High-pressure pretreatment also increased the production of protein hydrolysates from chicken bones [61].

Collagen is another important ingredient that can be isolated from meat by-products. This can be achieved through an alkaline procedure, and then, biomodifications can increase its digestibility. Prokopová et al. isolated collagens to produce gelatins from broiler chicken stomachs using a mixture of 0.2 mol/L NaCl, 0.06 mol/L NaOH, and petroleum ether and ethanol [68]. Lukin prepared high-quality lyophilized collagen hydrolysate using protepsin. Subsequently, the produced collagen had great practical importance in the sausage formulation, obtaining the best results at 15% enrichment [69].

Meat by-products can be a source of flavoring for further food applications. Formulation and concentration of volatile compounds in flavoring supplemented with hydrolysate from goat and chicken were studied by [61,64]. The pH affected the aroma profile, resulting in a strong meaty flavor at pH 4 and sweet fatty flavor and a goat aroma at pH 6 [64]. Alternatively, protein fractions (soluble and insoluble extracts in sodium citrate buffer 0.1 M pH 5) obtained from porcine spleens were successfully employed as emulsifying (soluble protein) and thickening (insoluble) agents for sausage production [70]. Protein precipitates by pH shifting from chicken by-products were used to formulate edible coating, achieving fat uptake reduction during deep frying of chicken drumsticks [71]. The addition of dry powder of chicken feet and heads served as a feasible approach to enhance physicochemical characteristics and improve the overall quality of forcemeat formulations [72].

Whey is the primary dairy by-product composed of mainly water (94%), lactose, proteins, and fats. α-lactalbumin and δ-lactoglobulin account for 70–80% of all the proteins in whey mass. Other constituents include immunoglobulins, lactoperoxidase, bovine serum albumin, and bovine lactoferrin [73]. The two major methods used by the dairy industry to handle whey are fermentation and membrane separation (e.g., ultrafiltration). Pires et al. applied ultrafiltration (40–45 °C; 3.0–3.5 bar) to produce symbiotic kefir products from sheep’s and goat’s whey concentrates, obtaining a product with a high protein content (7.8–16.4%) [74]. Likewise, Pavoni et al. applied similar conditions to ovine whey [75].

Himashree et al. studied the substitution of water by different concentrations of whey (0–100%) in bread. It resulted in high protein and fat content, decreased acrylamide concentration, and improved textural properties [76]. Kusio et al. used whey protein concentrate (76.8%) to produce high-protein, fat-free dairy desserts. Rheological properties, appearance, and antioxidant activity were enhanced in the final product [73]. Similarly, Dinkçi et al. used whey protein concentrate to produce a probiotic beverage with high phenolic compounds and antioxidant activity [9]. The following (Table 1, Table 2, Table 3 and Table 4) provide deep details about the ingredient preparation and their application on various food products regarding protein.

### 2.2. Fiber

The importance of dietary fiber (DF) has been related to its numerous health benefits, such as constipation prevention, cholesterol and blood glucose reduction, and improvement of gut microflora [89]. The World Health Organization has also established their recommended daily intake as 25 g/day, but many people do not achieve such amounts [90]. Thus, researchers and companies have focused their attention on the formulation of fiber-rich food exploiting by-products as alternative and sustainable sources of DF.

Fruits and vegetables including legumes and cereals are the main sources of DF. DF can be distinguished into two groups based on their solubility in hot water: soluble and insoluble dietary fibers. Soluble fibers consist of non-cellulosic polysaccharides such as β-glucan, inulin, pectin, and resistant starch, whereas insoluble fibers include cellulose, hemicellulose, chitosan, and lignin [91]. Peel, seeds, and pomace from different fruits (e.g., banana, apple, grape, tomato, and pepper) and vegetables (e.g., carrot, onion, and lettuce) are commonly used by-products, followed by meals from nuts, cereals, and oilseeds obtained after fat extraction and spent brewer’s yeast as a by-product of beer production (Table 5). DF is a very heterogenous group; therefore, their processing and food origin may influence their functionalities both in terms of physicochemical and fermentable properties [90]. Most by-products are minimally processed to obtain a shelf stable powder; thus, freeze or air drying are applied to stabilize the by-product and further grinding and sieving to achieve a homogeneous powder [29,92,93,94,95,96]. In some cases, disinfection and/or blanching have been necessary to ensure microbial safety and enzyme inactivation prior to drying [25,97,98,99]. Instead, defatted meals from hempseeds [100], walnut and sesame [15,101], durum wheat [22], and pepper seeds [28] were directly ground. A few simple steps are sufficient to convert a by-product into a fiber-rich food ingredient with a fiber content varying from about 10% to 65% depending on the type of plant source.

Additionally, fiber fractions (e.g., pectin, cellulose, and β-glucans) may be extracted, obtaining a purer ingredient with a high commercial value [102]. Acid extraction is commonly used to extract pectin from agro-industrial by-products. The powdered by-product is dispersed into water, and then, pH is adjusted to 1.5–2 and heated to 80–90 °C for 45 min up to 3 h. Insoluble material is removed by filtration, and the rest is dispersed into 70–95% ethanol with a sample-to-solvent ratio of 1:2–1:4 to induce pectin precipitation. Afterward, the precipitate is collected, dried, ground, and sieved [103,104,105]. As a green alternative to the use of solvents, US has been employed to extract pectin from celery root peel [102]. The by-product dispersed into water was subjected to US treatment (40–60 °C for 10–30 min) with a power level between 40% and 60% to improve extraction efficiency in a shorter time. After that, water-soluble pectin was extracted by distillation, with 75 °C as the chosen optimal temperature to ensure enzyme inactivation and the bioavailability of nutritional compounds.

Cellulose was obtained from oil palm empty fruit bunch after several acid treatments (pH 4) with acetic acid addition to the by-product dispersed in water and sodium chloride, for about 2 h at 70–80 °C. The solution was rinsed, dried, and extracted with alkali for 2 h at room temperature [106]. Another study used a harsh alkaline treatment (pH 12) to degrade the strong cell wall of spent brewer’s yeast and solubilize its components, which are mainly β-glucans and mannoproteins, often covalently linked together [19].

Fiber-rich ingredients and fiber fractions are often used in the food industry not only to improve the nutritional quality of food but also because of their potential functional properties, such as water-holding capacity and emulsifying and gelling capacities. These ingredients have been mostly employed for the development of pasta, bread, and other bakery goods with or without gluten. Pasta was enriched with 5% to 30% of fiber-rich ingredients, and it also fulfilled the claim “source of fiber” when ≥10% was replaced with hempseed flour containing 46% of dietary fibers [100]. The addition of ≤10% fiber-rich ingredients allowed to improve nutritional and technological quality, while exceeding this amount may negatively affect sensory quality and promote cooking loss [94,100]. In gluten and gluten-free bread, an enrichment level of 3 to 12% provided a higher amount of nutrients and fermentable sugars to yeasts, thus resulting in higher CO_2_ production and dough development [96,103,107]. In turn, the higher the substitution level, the weaker the gluten network [108]. Other bakery goods like biscuits, muffins, and macarons were enriched up to 20% [98], 30% [25], and 50% [101], respectively. However, they were mostly considered acceptable when the substitution level did not exceed 10% [25,98,101,109]. It is noteworthy that the higher the fiber content, the darker and harder the final products. The substitution of starch with fibers (or proteins) determines a reduction in the expansion volume and the porosity, thus resulting in a harder texture. This was highlighted also for the formulation of extruded and freeze-dried snacks [29,99,110,111].

The gelling, emulsifying, and water-binding capacities of fibers are suitable also for the development of emulsions and semi-solid foods. Stable and viscous products were obtained by adding both powder ingredients at 2–6% enrichment [15,18] and liquid ones at 20–35% [19,31] in emulsions or 15–62% in a yogurt-based beverage [102]. By contrast, a high enrichment level may strongly affect the odor, thus reducing consumer acceptability.

Fiber derived from fish by-products is becoming increasingly popular due to its potential benefits, environmental sustainability, and various applications. This type of fiber is mostly derived from fish skin and bones [45,46] or from shellfish shells [44], where chitin and chitosan (chitin-derived fiber) are obtained. Recently, the application of chitosan in the food industry has been focused on the synthesis of biodegradable film or coating material with valuable properties such as biodegradability, low toxicity, and strong antimicrobial and antioxidant potential [44,88]. In oenology, chitosan originating from shrimp and crab shells was tested for wine clarification as a potential replacer of fungus-derived chitosan [112].

The three primary stages of chitin extraction to obtain chitosan from crustaceans are pre-treatment, deacetylation, and posttreatment. The pre-treatment includes raw material washing and milling. This step is targeting the removal of mineral using acid washing and the removal of protein, glycoprotein, and branching polysaccharide using alkaline washing. In the deacetylation, the acetyl group from the chitin chains is removed using a high concentration of alkaline at high temperatures for a short time. In the posttreatment, recovery of deacetylated chitin (i.e., chitosan) takes place using low-concentration acid, and the chitosan is further precipitated at pH 10, washed, and dried [112]. Nonetheless, there are some variations in the literature regarding alkaline or acid concentrations, type of organic acids used, temperature, and time. The most challenging step is to avoid the use of environmentally unfriendly deacetylation procedures such as the enzymatic approach since the reaction of deacetylase requires long time. Different cases are detailed in the literature, with a description of each extraction step and its impact [113].

For example, four different organic acids (malic, acetic, succinic, and hydrochloric acid) were used to dissolve (1% *v*/*v*) commercially available chitosan from shellfish by-products for further use in wine sedimentation. However, all dissolved chitosan solutions showed great and fast wine clarification compared to the conventional chitosan from *Aspergillus niger* culture [112]. In another study, Asian tiger shrimp (*Penaeus monodon*) shells were dried in a hot air oven and milled before chitosan extraction. In detail, the hydrolysate powder was demineralized for 24 h at room temperature while being continuously stirred using a solid-to-solvent ratio of 1:10 (*w*/*v*) and 1 M HCl. A paste was produced after centrifugation at 5000 rpm, neutralization, and overnight drying at 45 °C. Afterward, chitosan was obtained by a deacetylation step using 60% NaOH (*w*/*v*) [44]. One gram of the extracted chitosan was employed to create a biodegradable packaging film, which was then applied to mackerel fish samples and showed a strong ability to postpone the fish samples’ oxidation. A high-quality edible coating material was obtained by a composite of chitosan–collagen, and it extended red porgy fillet shelf life, as reported by [88]. As noticed, the main use of fiber components from fish by-products concerns are non-dietary (i.e., chitosan). Thus, their main application in the food sector is in the field of edible films and food packaging materials [88]. The following (Table 5 and Table 6) provide deep details about the ingredient preparation and their application on various food products regarding fiber.

**Table 5:** Fruit and vegetable processing by-products with fiber benefits and their food applications.

By-Products	Ingredient Preparation	Food Application	Properties	References
Grape pomace	Addition (or not) of lyoprotectors → freeze drying → grinding	Gluten-free cookies (10–20% enrichment) Filling (17.2 g pomace flour)	↑ Fiber, protein, fat; ↑ sensory properties	[114]
Carrot pomace	Air drying (60 °C, 12 h) → grinding (250 mm)	Muffins (5–20% enrichment)	↑ Crude fiber, ↑ firmness, ↑ taste, ↑ appearance (10% the best sensory results)	[109]
Coconut residue	Drying → grinding	Spread (16% enrichment)	↑ Protein, fiber, good spreadability, ↑ sensory acceptability	[92]
Banana peel	Disinfection → drying (63 °C, 24 h) → grinding (0.8 mm)	Biscuits (10–20% enrichment)	↑ Fiber (+20% at 10%), ↑ dark color, good sensory acceptability (10% maximum)	[98]
		Bread (10% enrichment)	↑ Fiber (+12.5%)	
		Pasta (5–10% enrichment)	↑ Fiber (+71.4% at 10%)	
Hemp seed meal	Fat extraction (cold press) → grinding	Gnocchi (5–20% enrichment)	↑ Fiber (source of fiber claim, >10%), ↑ cooking loss, ↓ cooking resistance, ↓ sensory acceptability (bitter taste, odor)	[100]
Pineapple peel, banana peel, and pumpkin seed	Banana, pineapple: cutting → sanitation → drying (60 °C, 8 h) → grinding Pumpkin seed: roasting (15 min) → grinding	Gluten-free muffins (17% enrichment)	↑ Fiber, good sensory acceptability (100% pineapple peel; 50% pineapple peel; 50% pumpkin seed)	[97]
Grape pomace and skin	Grape by-products obtained commercially	Frankfurt and Spanish sausage (0.5% grape skin, 3–6% inulin, 0.5–1% β-glucan)	↓ Fat reduction, ↓ boar taint, ↓ brightness, ↓ hardness	[115]
Male date palm flowers	Drying (40 °C, 24 h) → grinding	Biscuits (3–9% enrichment)	↑ Fiber (source of fiber claim, >6%), darkness, hardness, good sensory acceptability (6% maximum)	[93]
Walnut oil cake	Fat extraction → grinding (0.8 mm)	Macarons (10–50% enrichment)	↑ Fiber, TPC, antioxidant capacity; ↓ sensory acceptability (10% maximum) ↓ costs	[101]
Brewery bagasse	Cooking (20 min, 100 °C) → filtration (residue) → drying (60 °C) → grinding (1 mm)	Bread (6% enrichment)	↑ Fiber	[80]
Jackfruit core	Pectin extraction: drying (60 °C, 20–24 h) → grinding → water dispersion (1:29) → acid extraction (pH 2, 80 °C, 105 min) → filtration → ethanol precipitation → drying (45 °C, overnight) → grinding	Vegetable soup	↑ Sensory properties	[105]
Tomato seed and skin, peppers, placenta	Drying (30–40 °C, 17 h) → grinding (0.25 mm)	Bread (3–12% enrichment)	↑ Fiber, WAC, dough development, softening; ↓ stability time, ↑ redness and yellowness	[96]
Celery root peel	Grinding → water dispersion (1:5) → homogenization → US extraction (25 kHz frequency, 40–60 °C, 40–60% power level, 10–30 min) → distillation (75–85 °C, 1–6 h) → refrigeration (4 °C)	Ayran Turkish beverage (15–62% enrichment)	↑ Pectin, sensory and structural properties; ↑ flavor, WAC, viscosity; ↓ color, phase separation; ↑ shelf life	[102]
Sugar beet pulp	Sucrose extraction in water (75 °C, 45 min) → filtration → cooling → bleaching (pH 11, 120 min) → fiber extraction (pH 6.5) → saponins removal (water: ethanol dispersion 1:4, overnight) → pectin removal (pH 1.6, 90 °C, 3 h) → filtration → ethanol dispersion (1:4, pH 3, 24 h) → filtration, rinsing → boiling (20 min, pH 6.5) → air drying → grinding (1 mm)	Gluten-free muffins (0.2–0.4% enrichment) depectinized or not-depectinized ingredient	↑ Viscosity, hardness (with pectin); ↑ fiber, swelling capacity, water and oil binding capacity (w/o pectin)	[104]
Paprika, pitted pepper, tomato pomace	Not specified	Bread (3–12% enrichment)	↓ Gluten network strength	[108]
Carrot pomace and beetroot– apple (7:3) pomace	Drying (40 °C) → grinding (0.4 mm)	Pasta (10–30% enrichment)	↑ Fiber, ↑ WAC (carrot pomace), ↑ technological and sensory (color, texture) properties	[94]
Apple and blackcurrant pomace	Obtained commercially	Freeze-dried snacks (2% enrichment)	↓ Porosity, hygroscopicity; ↑ brittle and fragile texture, color changes	[110,111]
Non-compliant broccoli and carrots and pomace	Washing → cutting → steam-blanching → (juice extraction) → freeze drying → grinding (0.8 mm)	Extruded snacks (3% enrichment with pomace, 20–100% enrichment with whole vegetable powder)	↓ Starch content, ↑ fiber, ↓ phenols, ↓ expansion volume	[99]
Orange and apple pomace, tomato peel, pepper peel, prickly pear peel, and prickly pear seed peel	Drying → grinding (0.5 mm) → pectin extraction: acid extraction (2 g sample +40 mL HCl, 90 °C, 45 min) → filtration (removal of insoluble material) → pectin precipitation: 95% ethanol dispersion (1:2), overnight → pectin purification: 70% ethanol dispersion (x2) → collection of the precipitate → drying (65 °C)	Gluten-free bread (2.5–7.5% enrichment)	↑ Dough height, ↑ CO_2_ production and retention, ↑ specific volume	[103]
Pea	Pea fiber obtained commercially	Bread (3–7% enrichment)	↑ Fermentation, ↑ CO_2_ production	[107]
Oil palm empty fruit bunch	Cleaning → drying (60 °C, 24 h) → grinding (0.2 mm) → α-cellulose extraction: NaCl dispersion + acetic acid (1:50, pH 4, 70–80 °C, 2 h, x 5) → rinsing→ drying (60 °C, overnight) → NaOH dispersion (1:25, 2 h, room T °C) → filtration→ washing→ drying (60 °C, 24 h) → carboxymethyl cellulose extraction: aqueous-alkaline dispersion (1:2), isopropyl alcohol addition (1 h, room T °C) → monochloroacetate addition (45 °C, 3 h) → pH adjustment with glacial acetic acid (neutral) → soaking in methanol (to remove impurities) → purification in ethanol	Low-fat ice cream (0.3% enrichment)	↓ Viscosity, hardness; ↓ overrun	[106]

‘→’ followed by, ‘↓’ low, ‘↑’ high.

**Table 6:** Fish processing by-products with fiber benefits and their food applications.

By-Products	Ingredient Preparation	Food Application	Properties	References
Shrimp and crab shells	Chitosan provided commercially with a deacetylation degree of 80–95%	Wine clarification	↑ Faster and greater sedimentation	[112]
Asian tiger shrimp (*Penaeus monodon*) shells	Shells drying → milling (710 µm) → chitosan extraction → demineralization (1 M HCl) → centrifugation → deacetylation: chitin conversion to chitosan using 60% NaOH (*w*/*v*) Chitosan film: 1 g chitosan + water (containing 1% acetic acid) → glycerol (plasticizer) → protein hydrolysates → drying	Biodegradable film	Excellent lipid oxidation-delaying capacity	[44]

‘→’ followed by, ‘↓’ low, ‘↑’ high.

### 2.3. Bioactive Compounds

A bioactive compound is a substance with a positive or negative biological activity in the organism depending on the type, the dose, and the bioavailability of the substance [116]. Plants (e.g., fruit, vegetables, cereals, and legumes), animals, fish, and microorganisms contain various bioactive compounds with proven biological activities such as antioxidant, anti-inflammatory, anti-diabetic, and antitumor ones; therefore, a sufficient intake may help to prevent or treat diseases [117].

Phenolics (e.g., phenolic acids, flavonoids, tannins, carotenoids, stilbenes, and lignans), terpenoids (e.g., tocotrienol, tocopherols, carotenoids, limonoids, and phytosterols), alkaloids, nitrogen-containing compounds, and organosulfur compounds are among the bio-active compounds mostly found in plants, including fruit, vegetables, cereals, and legumes [118]. Many researchers have focused their attention on the recovery of bioactive compounds, especially phenolics, from fruit and vegetable by-products for food enrichment both to face environmental issues and to meet consumers’ needs for healthy choices. For this purpose, various by-products have been already exploited, such as peel, stems, leaves, pomace, pods, press-cakes, as well as non-compliant fruit and vegetables.

Many studies added the by-product directly in the liquid or powder form into the food formulation without applying any isolation and purification of bioactive compounds. In general, by-products are washed, disinfected, cut, and blanched. Blanching is needed to inactivate enzymes that may degrade and oxidize bioactive compounds, such as polyphenol oxidase, peroxidase, and myrosinase. Hot water (98–100 °C for 1–5 min) or steam blanching are commonly used [24,25,99,119,120,121]; however, some authors demonstrated the potential of microwaves [122,123]. Later, by-products are stabilized by air drying or freeze drying or even sun drying, as reported by Traffano-Schiffo et al., and then ground to a fine powder [124]. The particle size of powders may affect not only the techno-functional properties of the ingredient when it is wholly included in the food formulation but also the extraction efficiency of bioactive compounds [119]. Depending on the extraction method, it is important to consider different parameters including but not limited to sample-to-solvent ratio, type of solvent, food matrix, treatment time, and temperature [118,121]. Novel extraction methods such as ultrasounds, microwaves, and high pressure (HP) have gained popularity in this field because of higher extraction efficiency and their lesser use of hazardous solvents and energy than conventional ones, which include, for example, Soxhlet, magnetic stirring, shaking, or maceration [118].

No toxic solvents such as ethanol and water, at different ratios from 0% [121,125] to 96% ethanol [126,127], are suitable for the extraction of hydrophilic compounds such as phenolics and glucosinolates, whereas deep eutectic solvents are a potential alternative to harmful solvents (e.g., hexane, acetone, and petroleum ether) for carotenoids extraction [128]. The sample-to-solvent ratio must be adequate to avoid saturation effects as well as the waste of solvents. Most sample dispersions were prepared in a ratio of 1:10–30, with few exceptions [102,126,129,130,131]. By contrast, 1:4 was found to be the optimal liquid/liquid ratio to obtain a phenol-rich extract from olive mill wastewater, using ethyl acetate as solvent [132].

As regards time and temperature, conventional methods usually take longer (up to 5 days) [133] and apply high temperatures, near 80 °C [131]. In general, the higher the temperature, the shorter the time. However, long extraction times and high temperatures are deleterious for thermolabile bioactive compounds [134]. On the other hand, novel technologies allow to reduce time and temperature to a maximum of 2 h and 60 °C [102,128]. In the case of ultrasounds, low frequencies (20–40 kHz) are commonly used to improve extractability. Traffano-Schiffo et al. showed that a high-intensity ultrasound (500 W) increased the extraction of phenolics from cowpea pods [124]. High-pressure processing (300–600 MPa) was also successfully applied for the extraction of lycopene from tomato by-products to enrich extra-virgin olive oil, directly used as a green solvent [135]. The results were very promising, and the pressure seemed to not affect carotenoid recovery. After treatment, the extract rich in bioactive compounds is separated from insoluble material by filtration or centrifugation and then stabilized either by drying or freezing.

A wide range of food products have been enriched with bioactive compounds, from bakery and pasta products to snacks, beverages, and emulsions. Most of the authors developing bakery and pasta products used the whole by-product powder with an enrichment level varying from 2.5% [136] to 15% [137] for pasta and from 5% [24,138] to 50% [101] for bakery goods. However, 10% is usually the maximum to obtain a product with good technological properties and sensory acceptability. The food matrix helps to prevent the thermal degradation of bioactive compounds during cooking [121]. Another strategy to protect these compounds is encapsulation. Kaderides et al. encapsulated pomegranate peel polyphenol using orange bagasse fibers as a carrier agent for spray drying and obtained cookies with a higher phenolic content and antioxidant activity despite bitter flavor and astringent taste [139]. Moreover, encapsulated systems, e.g., microemulsions [140], nanoparticles [141], and hydrogels [124,142], allowed the increase in bio-accessibility of bioactive compounds. Similarly, emulsion-based foods such as yogurt [102,143], butter [144], dressing [145], ice cream [146], and mayonnaise [133] registered an increase in bioactive content, bio-accessibility, and antioxidant activity, in some cases improving also color and flavor.

Bioactive-rich extracts have been widely applied for liquid food formulations such as instant drinks [120] and soups [95], fermented soy milk [147], kombucha [79,148], smoothies [131], distilled spirits [149], wine [150], and beer [151], with the aim to improve the nutritional quality and antioxidant activity of the final product. The antioxidant potential of bioactive compounds, mainly phenolics, has been exploited to enhance the oxidative stability of vegetable oils from rapeseed [130], soybean [129,152], olive [135], sesame [153], and sunflower [132].

Valorization of fish by-products for their bioactive compounds focuses mainly on extracting high-quality oil rich in nutrients, especially polyunsaturated fatty acids such as EPA and DHA, which have been recommended to be included in a routine diet to maintain good health. These bioactives from fish by-products are characterized by antioxidant, anti-obesity, antitumor, anti-inflammatory, and anti-allergenic activities as well as a protective action against cardiovascular disease. Bioactive oil is usually extracted from the side streams of fish by-products, including backbones, heads, gills, and viscera. These by-products may be subjected to freeze drying prior to oil extraction [154,155,156,157]. Differently, several by-products are processed wet [156,158,159]. Occasionally, by-products are submitted to a cooking step at 95 °C for 12 min before pressing [160].

Food-grade solvents (i.e., hexane and ethanol) are widely used for oil extraction. Some traditional methods are applied, such as Soxhlet [158,159] or the Bligh and Dyer method [156]. Alternatively, some mild processes include US, microwave-assisted extraction (MAE), pressure extraction, and super-critical fluid extraction [155,156,157,161].

Different studies have reported the effects of raw material treatment and extraction methods on the oil yield and its nutritional characteristics. For example, Kalogianni et al. reported the highest antioxidant activity measured by DPPH and ABTS assays in oil extracted using supercritical fluid extraction starting from freeze-dried samples [156]. The authors performed the extraction at 37 °C and 20 MPa pressurized with CO_2_ for 30 min with a flow rate of 3 mL/min. Their findings also highlighted significant amounts of total PUFA (20.7%) represented mainly by EPA and DHA with a considerable oil yield [156]. The use of MAE for freeze-dried by-products of salmon allowed the recovery of >60% of oil in <19 min [155,157]. MAE oil was characterized by a healthy lipid profile due to the high content of EPA and DHA as well as high antimicrobial and anti-inflammatory properties [155]. High-quality crude oils were produced by pressing cooked by-products of tuna, seabass, seabream, and wild sardines. The oil derived from tuna, seabass, and seabream had low levels of anisidine value, peroxide, and free fatty acid content. Seabass and sea-bream oil had the highest α-tocopherol content, whereas sardine oil had the highest concentration of EPA and DHA (32.8%) [160].

Furthermore, fortifying food products with omega-3-rich oil offers the potential to scale up and valorize the use of fish by-products. For instance, a functional flavored yogurt was incorporated with fish by-product oil derived from Nile perch, containing 20.5% omega-3 fatty acids (consisting of 3% EPA, 6.2% DPA, and 9.0% DHA) [162]. Consequently, the target daily consumption of omega-3 fatty acids was met, and there was no observable impact of the fish oil on flavor [162]. Similarly, pork sausage was fortified with oil extracted from skipjack tuna heads and viscera. The overall acceptance, ABTS+, DPPH radical scavenging activity, and antimicrobial properties of the sausage were improved, with significant increases in EPA (4.15%) and DHA (21.96%) [154]. Deep-fried chips enriched with fish by-product oil and artichoke bract powder had a booster in their nutritional quality. They showed reduced triglycerides, cholesterol, and LDL levels, along with increased average lipid content and essential fatty acids [158]. The incorporation of fish oil and artichoke powder also effectively improved their vulnerability to oxidation. These findings are likely due to the capacity of these enriched chips to block lipid peroxidation, enhance antioxidant status, and obstruct lipolysis and the subsequent insulin-induced inhibition of free fatty acid release. Thus, reducing the associated risk of cardiovascular diseases [158].

Regarding dairy by-products, whey is a source of protein with high biological value as well as branched chain amino acids, essential amino acids, and high-sulfur-containing amino acids, which support antioxidant activity. They promote glutathione synthesis, enhance probiotic bacteria growth, and protect from oxidative stress due to their antioxidant effect [9]. The application of whey for antioxidant purposes was studied by Alwohaibi et al. by valorizing different dairy by-products. The study was focused on using sweet whey, sweet buttermilk, and skimmed milk to produce a functional dairy–millet beverage fermented with *Lactobacillus paracasei* as an adjunct culture [163]. Four fermented milk beverages (water-based, whey-based, buttermilk-based, and skimmed-milk based) were developed and analyzed for several characteristics, including their antioxidant properties. All fermented milk beverages produced using the dairy by-products exhibited significant antioxidant properties in comparison to the water-based fermented beverage. Additionally, all samples had a low glycemic index and glycemic load [163]. The following Table 7, Table 8 and Table 9 provide deep details about the ingredient preparation and their application on various food products regarding bioactive compounds.

## 3. Conclusions

The increasing demand for more nutritious, healthy, and sustainable food products has moved the attention of researchers and companies to find alternative sources of macro- and micro-nutrients, especially proteins, fibers, and bioactive compounds. Food processing by-products from animal, fish, and vegetable origin represent a valuable alternative. The effective use of by-products can help to increase resource efficiency, lower the burden of waste disposal, and produce useful inputs for the food industry. Besides conventional extraction methods, novel technologies may be adopted to produce functional high-quality ingredients in the form of powders or liquid extracts. Protein-, fiber-, and bioactive-rich ingredients allow the improvement of the nutritional quality of fortified food. Among them, bioactive compounds (e.g., phenols, carotenoids, omega-3 fatty acids, etc.) can both provide health benefits for the organism and increase the oxidative stability and the shelf life of the final food. Depending on the type of food, proper enrichment levels should be carefully evaluated to obtain the optimal physicochemical properties.

## Figures and Tables

**Table 1:** Fruit and vegetable processing by-products with protein benefits and their food applications.

By-Products	Ingredient Preparation	Food Application	Properties	References
Artichoke bracts and tomato	Non-thermal patented treatment (Patent n°001426984) → freeze drying	Fresh pasta (3% enrichment)	Good protein network, ↑ sensory acceptability	[77]
Pepper placenta, pepper defatted seeds, tomato seed/skin	Air drying (17 h, 30–40 °C) → fat extraction (from seeds, solvent) → grinding	Pasta (10–30% enrichment)	↑ Protein, fiber	[28]
Broccoli leaf	Blanching (hot water, 1 min) → freeze drying → grinding	Pasta (2.5–5% enrichment)	↑ AA composition, ↑ cooking loss	[23]
Broccoli leaf	Blanching (hot water, 1 min) → freeze drying → grinding (0.6 mm)	Gluten-free bread (5% enrichment)	↑ Protein, minerals, ↑ antioxidant activity and anti-AGE activity, ↑ specific volume, bake loss	[24]
Cauliflower stems, leaves, stalks	Blanching (2–3 min) → drying (60 °C, 16 h) → grinding (0.8 mm)	Muffins (10–30% enrichment)	↑ Protein, fiber, TPC, TFC, antioxidant activity, ↑ dark color, ↑ hardness, ↓ specific load volume, chewiness, springiness, cohesiveness, ↓ sensory acceptability (max 10%)	[25]
Mushroom stems	Freeze drying → grinding (0.25 mm) → ergosterol removal: dispersion (3:100) in 96% ethanol (residue) → drying (30 °C, 72 h)	Spray-dried emulsions (5% enrichment)	↑ Viscosity, stability. ↑ yield, ↓ oxidation	[78]
Broken cashew kernels	Fat extraction (solvent) → grinding (0.25 mm) → water dispersion (1:15) → isoelectric precipitation (pH 3–4.5, 15 min) → drying	Vegan burgers (27% replacement)	↓ Solubility, good sensory acceptability	[30]
Black gram by-product	Milling → fractionation (0.4–0.8 mm)	Extrudates	↑ WAC, ↑ starch digestibility, ↓ phenolics (flavonoids, proanthocyanidins, saponins)	[26]
Quinoa okara	Water dispersion (1:10) → alkaline extraction (pH 9, 30 min, 55 °C) → US precipitation (75% amplitude, 30 s on—15 s off, 10 min) → air drying	Emulsions (2–6% enrichment)	↑ Protein, fiber, ↑ ES, ↓ particle size	[18]
Soy okara	Drying (100 °C, 4 h) → grinding (0.3–0.6 mm)	Gluten-free extruded snacks (8–40% enrichment)	↑ dark red color, ↓ expansion, ↑ nutritional/sensory quality (29–40%)	[29]
Aquafaba	Chickpea: broth, 1:2 (*w*/*v*) → cooking (100 kPa, 35 min) → freezing (liquid)	Meringue (egg white replacement)	↑ Foaming and emulsifying properties, hardness, fracturability, similar color, ↓ alveoli	[32]
Aquafaba	Pre-soaking: chickpea: water, 1:3 (*w*/*w*) (5 °C, 16 h) → Cooking: chickpea: water, 1:2 (*w*/*w*) (118 °C, 70–80 kPa, 30 min) → filtration → freezing	Emulsion (25–35% replacement)	↑ Gel-like behavior, ↓ cost	[31]
Aquafaba	Soaking (chickpea: water, 1:4, *w*/*v*, 8–10 h) → cooking (chickpea: water, 1–5, *w*/*w*, 45 min) → cooling → filtration (liquid) → freezing	Whipped cream (egg white replacement)	Good egg white replacer, ↑ foam particles, ↓ foam stability, ↓ hardness, gumminess	[79]
Walnut cake and sesame cake	Fat extraction → grinding	Low-fat mayonnaise (2–4%)	↓ Carbohydrate, ↑ protein, fiber, ↑ ES, ↓ hardness, adhesiveness, ↑ sensory properties	[15]
Soybean extruded-expelled meal	Water dispersion (1:10) → sterilization (15 min, 120 °C) → fermentation → freeze drying	Gluten-free bread (6% enrichment)	↑ Protein, fiber (cellulose lignin), ↑ specific volume, ↑ iron bio-accessibility, ↑ crumb color, ↑ aeration	[13]
Durum wheat cake	Fat extraction (solvent) → air classification (grader wheel speed 3000 rpm, airflow 50 m^3^/h)	Snack protein bar (10–50% enrichment)	↑ Protein and fiber, ↓ fat, ↑ firmness, ↑ yellowness, ↑ caramel flavor	[22]
Coconut oil by-product	Fat extraction (cold press) → grinding → water dispersion (1:10) (60 °C, 1 h) → homogenization (10,000 rpm, 5 min) → US (405 W, 2.52 min) → filtrated liquid	Full-fat and low-fat vegan ice-cream (1–3% enrichment)	↑ Protein and fat, ↓ cost	[20]
Peanut meal	Fat extraction (press)	Gluten-free snacks (26% enrichment)	↑ Protein, ↑ shelf life, ↑ crispiness	[14]
Rapeseed press cake	Fat extraction (cold press) → water dispersion (1:9) → alkaline extraction (pH 10.5, 4 h) → isoelectric precipitation (pH 3–6.5) → freeze drying	Emulsions (0.05–5%)	↑ ES (pH 5–6), ↑ protein yield (pH 4.2), ↓ droplet size (pH 6–6.5)	[17]
Spent brewer’s grain	Cooking (20 min, 100 °C) → filtration → homogenization → pasteurization (5 min, 80 °C)	Protein beverage	↑ Protein	[80]
Spent brewer’s yeast	Vacuum drying (40 °C, −0.7 bar) → grinding → water dispersion (1 h) → alkaline treatment (pH 9–12, 20–25 °C, 2 h)	Emulsions (20% enrichment)	↑ Mannoprotein solubility, ↑ viscosity, ↑ structure stability, color changes	[19]

‘→’ followed by, ‘↓’ low, ‘↑’ high.

**Table 2:** Fish processing by-products with protein benefits and their food applications.

By-Products	Ingredient Preparation	Food Application	Properties	References
Tilapia head	Removal of non-collagenous protein and fat with 1 mol/L of NaOH → mineral removal with 0.2 mol/L of EDTA → different extraction methods: (a) Hot water-pretreated gelatin (HWG) (b) Acetic acid-pretreated gelatin (AAG) (c) Sodium hydroxide-pretreated gelatin (SHG) (d) Pepsin enzyme-pretreated gelatin (PEG)	Fish oil emulsions with gelatin solutions (2–10 mg/mL)	(1) Gel strength: PEG > AAG > HWG > SHG (2) Foaming properties, emulsion viscosity, emulsion activity, and emulsion stabilization ability: PEG > HWG ≥ AAG > SHG	[45]
Fermented fish bone residue (FFBR)	Alkaline treatment (NaOH at 3–6%) and three soaking times (0, 1, and 2 h)	Fish sausage fortification with properly treated bio-calcium (0–36 g)	↓ Salt content and intensity of fermented fish smell ↑ Calcium and phosphorus contents	[33]
Seabass (*Dicentrarchus labrax*) heads and bones	Hydrolysates production: alcalase (60 °C for 3 h/pH 7.6) → spray drying Fish meal production: steam boiling (20 min) → pressing → oven drying (60 °C for 30 h) → grinding	Biscuits (2.5–5% enrichment)	↑ Intensities of color, toasted, and fish flavors; nutritional enrichment in protein and PUFAs	[81]
Asian tiger shrimp (*Penaeus monodon*) shells	Hot air oven drying → US and NADES → subcritical water hydrolysis (150–230 °C, 3 MPa, 20 min) → filtration	Biodegradable film (2–6%) for mackerel fish samples	↑ Protein (391.96 mg BSA/g) obtained at 190 °C/UPT/NADES; average molecular size of protein molecules < 1000 Da	[44]
Salmon heads, fins, tails, and viscera	pH shifting: (2 mol/L NaOH) for 30 min/pH 11 → filtration → centrifugation → (2 mol/L HCl) pH 5.5 → precipitation → centrifugation → dialysis → freeze drying	Stabilized gels to encapsulate probiotic emulsions (4%, *w/v* complex protein solution; 2%, *w/v* alginate).	↑ Emulsifying ability and emulsion stability	[50]
Tuna *(Thunnus alalunga)* heads	Hydrolysates production: alcalase (60 °C for 3 h/pH 7.6) → spray drying Fish meal production: steam boiled (20 min) → pressing → oven drying (60 °C for 30 h) → grinding	Biscuits (2.5–5% enrichment)	↑ Protein and moisture	[35]
Canned sardine waste, mix of sardine meat, and by-products	Hydrolysates production: protamex, 0.50% → incubation (50 °C, 2.5 h) → addition of 0.7%; flavourzyme → adjusted to 15% salt and 14% *Aspergillus oryzae koji* → fermentation	Fermented fish sauce (50–100%)	No strong or unpleasant flavor, ↑ quality sauce	[38]
Tra fish (*Pangasius hypophthalmus*)	Washing (0.3% NaHCO_3_, 0.3% salt) → slicing → seasoning → grinding → balls forming → blanching (40 °C, 10 min → 2 cooking methods applied for 3–6 min, (1) steaming (100 °C) (2) blanching (80–100 °C)	Fish balls	↑ Protein content, texture, color	[82]
Blue whiting (*Micromesistius poutassou*)	Protein isolate obtained commercially (92%)	Pasta (5–15% enrichment)	↓ Optimal cooking time, swelling index, and water absorption; ↑ cooking loss; ↑ protein content, firmness, and adhesiveness	[83]
Abdominal fat of striped catfish (*Pangasianodon hypophthalmus*)	Oil extraction: Abdominal fat washing and draining → chopping → heating (5 h, 70 °C → filtration → oil purification → microencapsulation by spray drying	Microencapsulated fish oil enriched with fish protein concentrates and chlorella powders	Mix of catfish oil, chlorella powder, and striped catfish protein concentrate 40%, 20%, and 40% had the best protein content (17.02%)	[52]
Fish bones and scales	Scales demineralization of gelatin: washing (10% *w/v* NaCl) → washing → treated with 2 M lactic acid solution → washed (yield of 76%) Flour: boiling bones for 30 min → drying (90 °C, 24 h) → grinding (0.6 mm)	High-protein financier and deep-fried puffed (5 g gelatin), panna cotta (6 g gelatin), whipped cream (24.6 g gelatin)	↑ Protein concentration	[84]
Nile tilapia *(Oreochromis niloticus*) and croaker *(Cynoscion virescens)*	Pressure-cooked → pressed → ground → drying → re-grinding	Instant food products and seasoned flours	↑ Protein content (55.41–39.86%) of Nile tilapia backbone and head flour	[58]
Parrotfish (*Chlorurus sordidus*) head	Enzymatic hydrolysis (chopped head mixed with water (1:2), neutral pH with 0.2 N NaOH → conditioning (50 °C, 5 min) → flavourzyme (0.2 AU/g) addition → incubation → inactivation/heat → centrifugation → spray drying	Yoghurt (0.1–3% enrichment)	Best enrichment (0.15%) with a viscosity value of 2.432 N·s/m^2^, syneresis 49.67%, WHC 37.4%, pH 4.36, TTA 0.76%, color L* 74.55, color a* −3.76, color b* 8.2	[41]
Pink perch (*Nemipterus japonicus*) head and viscera	Enzymatic hydrolysis (alcalase, 58 °C, 85 min → inactivation/heat → centrifugation → freeze drying → microencapsulation → freeze drying	Soup fortification (15% protein + 11% protein hydrolysate, microencapsulated protein hydrolysate (27%), and sun-dried whole fish powder (16%)	↑ Overall acceptability	[36]
Pink perch (*Nemipterus japonicus*)	Enzymatic hydrolysis (alcalase, 57.9 °C, 85.8 min, 0.15% (v/w) E/S ratio) → freeze drying → microencapsulation	Microencapsulation of surimi by-product	High yield (17%), good functional properties, and moderate antioxidant activity;↑ amount of essential amino acid (35%), ↓ bitterness and odor	[47]
Pangas (*Pangasius pangasius*)	Alkaline extraction and precipitation	Fish sausages (2.5–10% enrichment)	↑ Crude protein and lipid content, ↓ gel strength	[34]
Roe, milt, and liver of plaice (*Pleuronectes platessa*), herring (*Clupea harengus*), and cod (*Gadus morhua*)	Washing → mixing →cooking	Fish pate	↑ Acceptability, ↑ protein content (21%) in roe pate	[85]
Sea bass (*Dicentrarchus labrax*)	Dipped in saline solution 8% → dried (60 °C/24 h)	Pasta (10% enrichment)	↑ Protein, fat, fiber, EPA, DHA; ↑ nutritional availability after cooking	[54]
Sea bass (*Dicentrarchus labrax*)	Dipped in saline solution 8% → dried (60 °C/24 h)	Pasta (10% enrichment)	↓ Texture properties except for adhesiveness, ↑ Nutritional profile, low impact on the technological quality	[86]
Chub mackerel *(Scomber japonicus)* tail	Minced fresh tail offcuts	Ready-to-eat fish paste (3.5–61.1%)	Paste with 61.1%: ↑ overall acceptability, flavor, color, texture, crude protein; ↓ crude fat percentage	[87]
Carp skins (*Cyprinus carpio*)	Gelatin extraction: 2.6% NaCl → filtration → freeze dying Hydrolysate production: protamex, alcalase, neutrase and flavourzyme → enzyme inactivation → centrifugation → SDS-PAGE separation → freeze drying	Snack	↑ Nutritional value and antioxidant power, good sensory qualities	[39]
Sea bass (*Dicentrarchus labrax*)	Dipped in saline solution 8% → drying (60 °C/24 h)	Fresh pasta (10% enrichment)	↑ Nutritional values, ↑ protection against oxidation, ↑ ω-3 and ω-6, adequate sensory profiles	[55]
Atlantic salmon (*Salmo salar*) and cod (*Gadus morhua*) backbones	Enzymatic hydrolysis, direct protein extracts by thermal coagulation without enzyme	Emulsions	↑ Emulsion activity in the products based on direct protein extraction	[49]
*Abeo rohita* head	US enzymatic extraction: mincing → pH adjusted (2 M NaOH) → US → pH 7.5 (2 M NaOH or 2 M HCl) → protamex incubation → inactivation → centrifugation → freeze drying	Umami compounds	↑ Efficiency of extraction best results at degree hydrolysis (22.73%) and extraction yield (76.34%) of umami extract achieved with a liquid/solid ratio of 3 (v/w), US time 19.20 min, hydrolysis time 140.45 min	[43]
*Sardinella aurita*	Protein isolates by alkaline process: homogenization → adjusting pH 11.0 (2 N NaOH) → centrifugation → precipitation (2 N HCl) → centrifugation → adjusting pH 7 → spray drying	Stabilized microencapsulated corn oil	↑ Encapsulation efficiency, ↑ protection of corn oil, ↑ best ratio protein isolates and maltodextrin 1:4	[51]
Brown stripe red snapper (*Lutjanus vitta*) protein hydrolysate	Enzymatic hydrolysis by alcalase → freeze drying	Deep-fried battered squid with hydrolysate (2–6% and 8%)	↑ Hardness and crispness, ↓ fat content at 8%, ↓ oil binding capacity, maximum acceptability scores at 4%	[37]
Surimi by-products	Lyophilized by-product (25 g protein) → alcalase 2.4 L (270,000 U/g) and trypsin (200,000 U/g) → inactivate protease → centrifuge → freeze drying	Gel for silver carb surimi	↑ Delaying protein oxidation, ↑ initial gelation properties, ↓ loss in gelation and water-holding capacity	[48]
Silver carp (*Hypophthalmichthys molitrix*) fin	Hydrolysate by four enzymes (trypsin, alcalase, papain, and neutrase) → freeze drying	Bighead carp (*Hypophthalmichthys* *nobilis*) fillets fortified	Trypsin and alcalase hydrolysates had strong ABTS scavenging activity and inhibition of protein oxidation, ↓ lipid oxidation	[40]
Yellow fin tuna red (*Thunnus albacares*)	Hydrolysate by papain enzyme: E/S ratio and hydrolysis time as 0.34% and 30 min, respectively at 60 °C and pH of 6.5 → spray drying	Mayonnaise	(50%) Replacement of egg yolk with protein hydrolysate: ↑ desirability, ↑ quality of emulsion, ↓ particle size, ↑ oxidative and physicochemical stability	[42]
Tilapia, salmon, and tuna	Thawing → washing → cooking (BHT at 0.5 mg/kg) → draining → pressing → milling → oven (60 °C, 24 h)	Cereal bars (90% tilapia protein concentrate and 10% salmon or tuna protein concentrate)	No change in the nutrients, sensory and microbiological profile ↑ mineral matter	[57]
*Priacanthus arenatus*	1st Heating (180 °C, 100 min) → milling → 2nd heating (200 °C, 40 min) → milling	Fish nuggets (10–40%)	40% fish waste flour: ↑ protein, lipid, and ash ↓ carbohydrate ↑ hue angle, a*, and b* values ↑ overall liking and preference	[56]
Chinese long snout catfish skin, silver carp skin, salmon skin, and Alaska pollack skin	Soaking with 0.1 M NaOH (pH 7) → soaking in 0.05 M acetic acid (pH 7) → incubation (55 °C) → filtration → freeze drying	Fish oil-loaded gelatin-stabilized emulsions (2–10 mg/mL gelatin)	↑ Gel strength of catfish and silver carp gelatins than marine fish skin gelatins, ↑ amino acids, ↑ contents of hydrophobic amino acids, ↑ molecular weights	[46]
Blue shark (*Prionace glauca*) skin	Pepsin soluble collagen extraction: shark skin mixed with 0.1 M NaOH (remove non-collagenous proteins) → fat removal → extraction (0.5 M acetic acid + 0.2% pepsin) → centrifugation → dialysation → freeze drying	Chitosan–collagen composite coating for red porgy (*Pagrus major*) meat	↑ Deterioration indexes at a ratio of 1:0.8 chitosan/pepsin, soluble collagen, ↑ physicochemical indexes	[88]

‘→’ followed by, ‘↓’ low, ‘↑’ high.

**Table 3:** Meat processing by-products with protein benefits and their food applications.

By-Products	Ingredient Preparation	Food Application	Properties	References
Porcine spleens	Soluble proteins extraction (sodium citrate buffer 0.1 M, pH 5) → centrifuging→ spray drying → washing of insoluble fraction → Pasteurization (80 °C, 45 min)	Sausages	Good functional properties	[70]
Broiler chicken stomachs	Separation of organic matter (0.2 mol/L NaCl, 0.06 mol/L NaOH, 1:1 petroleum ether/ethanol) → conditioning of collagen (protamex, pH 6.5, 0.1–0.2 wt%) → hot water	Gelatins	↑ Gelatin yield (65%), gel strength (25–439 Bloom), digestibility (100%), melting point, ↓ ash	[68]
Trimmings, skin, meat-left-on-bone of chicken drumstick	Homogenization → alkaline extraction (pH 11.5, 10 min) → centrifugation → isoelectric precipitation (pH 5.5, 10 min) → centrifugation	Edible coating	↑ Moisture content, ↓ fat uptake, ↑frying yield in coated samples	[71]
Chicken bones	High pressure pretreatment (121 °C, 4 h) → oven drying (55 °C, 5 h) → enzymatic hydrolysis → Maillard reaction → autoclaving (113 °C, 10 min).	Flavoring agents	↑ Protein and lipid content	[61]
Chicken feet and head	Grinding → dispersion (1:1) in 10% solution of ascorbic acid → drying	Forcemeat	↑ Protein, ash content	[72]
Cattle lips and ears	Cleaning → crushing → enzymatic hydrolysis (protepsin, 1:2 E/S, 0.5–1.5 h, 17–22 °C) → filtration → freeze drying	Forcemeats (5–20% enrichment)	↑ Moisture-retaining capacity	[69]
Goat viscera	Cleaning → crushing → homogenization → enzymes inactivation (90 °C, 15 min) → cooling → enzymatic hydrolysis (alcalase, 0.8% E/S), 240 min) → inactivation (90 °C, 15 min) → cooling → Maillard reaction → homogenization → autoclaving (121 °C, 60 min)	Meat flavorings	↑ Meat aroma	[64]

‘→’ followed by, ‘↓’ low, ‘↑’ high.

**Table 4:** Dairy processing by-products with protein benefits and their food applications.

By-Products	Ingredient Preparation	Food Application	Properties	References
Whey	Heating of milk (70 °C, 20 min) → coagulation (1% citric acid) → filtration	Bread (0–100% water substitution)	↑ Protein, fat	[76]
Whey	Whey-protein concentrate (76.8%) obtained commercially	High-protein fat-free dairy desserts	↑ Texture and appearance, ↓ water activity	[73]
Sheep and goat cheese whey	Ultrafiltration (40–45 °C, 3.0–3.5 bar → pasteurization (65 °C, 30 min) → homogenization → freezing	Symbiotic kefir	↑ Protein concentration	[74]
Cow, sheep, and goat whey	Whey-protein concentrate (>60%) obtained commercially	Probiotic beverages	↑ Protein, free amino acids, TPC, antioxidant activity	[9]

‘→’ followed by, ‘↓’ low, ‘↑’ high.

**Table 7:** Fruit and vegetable processing by-products with bioactive benefits and their food applications.

By-Products	Ingredient Preparation	Food Application	Properties	References
Longan peel	Fat extraction → freeze drying → 80% ethanolic extraction → solvent evaporation	Soybean oil	↑ Oxidative stability during frying, tocopherols; ↓ peroxides, conjugated dienes, TBARS	[152]
Black currant pomace	Obtained commercially	Freeze-dried vegetable snacks (1–5% enrichment)	↑ TPC, antioxidant activity; ↑ dark color, ↓ water activity, ↓ porosity, ↑ hardness, crispiness	[164]
Pomegranate peels, olive oil pomace, broccoli root and leaves	Broccoli: drying (35 °C, 48 h) → grinding (0.5 mm) pomegranate: washing and disinfection → drying (50 °C, 48 h) → grinding (0.5 mm) olive: drying (35 °C, 72 h) → grinding (0.5 mm)	Fresh pasta (10% broccoli and 3–6% olive and pomegranate by-products enrichment)	↑ Phenols, flavonoids; ↓ microbial growth; ↑ shelf life	[165]
Pomegranate peels, olive oil pomace, broccoli root and leaves	Pomegranate, broccoli: washing → disinfection → cutting → drying (45 °C) → grinding (0.5 mm) Olive: drying (45 °C) → grinding (0.5 mm)	Fresh pasta (3–8% enrichment each)	↑ Phenols, flavonoids, antioxidant activity, shelf life (especially pomegranates)	[166]
Papaya peel	Washing → cutting → freeze-drying → grinding (<2 mm) → carotenoid extraction: dispersion (1:20) in THF+0.01% BHT+ magnesium carbonate →homogenization → US treatment (30 min) → centrifugation → re-extraction with acetone (pellet) → supernatant collection → diethyl ether addition → organic phase collection → filtration → vacuum drying	HPP-treated microemulsions (7 mg/g soybean/sunflower/coconut oil)	↓ Particle size, ↑ carotenoid bio-accessibility (sunflower, soybean oil)	[140]
Coconut husk	Washing → cutting → grinding → MW blanching (2450 MHz–900 W, 40–160 s) → cooling → grinding	Kheer dairy-based dessert (5–15% enrichment)	↑ Flavor, color (10% maximum)	[122]
Apple pomace	Drying → milling	Gluten-free bread (5–15% enrichment)	↑ Phenols, flavonoids, chlorogenic acid, phlorizin; ↑ antioxidant activity; ↑ sensory acceptability	[138]
Apple pomace	Freeze drying → UV sterilization	Fermented soy milk	↑ Phenols, ↑ antioxidant activity, ↑ viable cell count	[147]
Lemon peel	Grinding	Flavoring gel	↑ thermal stability ↑ safety, quality	[167]
Mango peel	Cutting → blanching (98 °C, 1 min) → drying (50 °C, 4 h)	Instant drink (2–6% enrichment)	↑ TPC, vitamin C, ↑ antioxidant activity, ↑ acidity	[120]
Pomegranate peel, orange juice residue	Pomegranate: drying (40 °C, 48 h) → grinding (0.1 mm) → water dispersion (1:32) → US treatment (50 W, 35 °C, pulse 7 s on—6 s off, 10 min) → homogenization with hydrated orange fibers (1:9) → encapsulation (spray drying)	Cookies (enrichment at 5000 ppm of phenolic concentration)	↑ Retention of encapsulated phenolics, ↑ antioxidant activity, ↑ bitterness, astringency, ↑ color, odor, ↓ texture	[139]
Banana, pineapple, squash, mango, calamansi, potato peels	Washing → cutting → drying	Distilled spirit (sample-to-solvent, 1:10, solvent: 40% ethanol/gin/vodka/tequila)	↑ TPC, DPPH (mango), gin/vodka good substitute for food-compatible solvents	[149]
Star fruit peel	Drying (40 °C, 24 h) → grinding → phenol extraction (sample: acetone, 1:20, 4–24 h, 30 °C, x2) > solvent evaporation	Sesame oil (200–1000 ppm)	↑ Oxidative stability during storage and frying (1000 ppm), good alternative to synthetic antioxidants	[153]
Non-compliant melon	Washing → peeling → deseeding → cutting → juice extraction → decantation (1 °C, 24 h, SO_2_ addition) → must	Melon-based wine	↑ TPC, antioxidants, ↑ fruity, floral aroma	[150]
Grape seed, olive stone	Flour obtained commercially	Crackers (10–30% enrichment)	↑ TPC, ↑ dark color, ↓ sensory acceptance, ↓ hardness (olive stone)	[168]
Olive mill wastewater	Filtration → liquid-liquid extraction with ethyl acetate (1:1–5, 4–70 °C, 0.25–24 h) → organic phase separation → solvent evaporation	Olive and sunflower oil (0.3% enrichment)	↑ lipophilicity and bioactivity of phenols, ↑ thermal stability, ↓ oxidation	[132]
Olive wastewater (oil + brine)	Centrifugation	Stuffing or paste for olives (63% enrichment oil fraction) and pickled vegetable (brine)	↑ Phenols, ↑ sensory properties	[169]
Olive leaf	Drying (T °C room, 15 d) → dispersion (1:10) in vinegar → homogenization → maceration (T °C room, 5 d) → filtration	Low-fat vegan mayonnaise	↑ Phenols (oleuropein), ↑ oxidative stability, ↑ shelf life, ↑ color, taste	[133]
Olive leaf	Washing → drying (120 °C, 8 min) → grinding → water dispersion (1:20→ US extraction → filtration (liquid extract) → freeze drying	Ripened sausage (0.02–0.08%)	↑ Weight loss, ↑ oxidative stability, ↓ hardness, ↑ microbial safety, natural additives (nitrite substitute)	[125]
Red onion peel	Washing → drying (40 °C) → grinding (1 mm) → UV sterilization → dispersion (1:14) in 70% acidified ethanol → US treatment (40 kHz, 25 °C, 40 min, 100 W) → centrifugation (liquid extract)	Ricotta cheese (1–3% enrichment)	↑ Phytochemical content, ↑ antioxidant activity ↑ texture, color	[170]
Onion skin	Washing → drying (50 °C, 12 h) → grinding (0.5 mm)	Pasta (2.5–7.5% enrichment)	↑ Phenol (quercetin, phenolic acids), ↑ antioxidant, anti-inflammatory activity	[136]
Onion skin	Washing → drying (T °C room, 14 d) → grinding (0.2 mm)	Extrudates (3–9% enrichment)	↑ Quercetin, ↑ TPC, antioxidant activity	[171]
Tomato by-products	Dispersion (1:10) in DES-LA (deep eutectic solvents/lactic acid, 1:2) → US (40 kHz, 30 °C, 1–2 h) → centrifugation (liquid extract) → extract: chitosan solution (40:60, *w*/*w*)	Edible films (40% liquid extract)	↑ Thermal stability, hydrophilicity; ↑ free radical scavenging rate, ↓ oxidation, ↑ shelf life	[128]
Tomato seeds and skin	Blanching → freezing → HPP extraction (by-product+ oil, 300–600 Mpa, 10 min) → agitation (45 °C, 45 min)	Extra virgin olive oil (10–20% enrichment)	↑ Lycopene recovery, good sensory acceptability (10%)	[135]
Tomato pomace, linseed meal	Tomato: grinding → drying (45 °C, 10 h) Linseed: cold pressing	Gluten-free pasta (10–15% enrichment)	↑ Fiber, lipid, tocols, phenols, ↑ antioxidant capacity	[137]
Artichoke bracts and tomato	Non-thermal patented treatment (Patent n°001426984) → freeze drying	Fresh pasta (3% enrichment)	↑ Phenols, antioxidant activity	[77]
Black carrot pomace	Freeze drying → grinding → UV sterilization → dispersion (1:10) in 70% acidified ethanol → US treatment (40 kHz, 25 °C, 40 min, 100 W) → centrifugation (liquid extract)	Yoghurt (1–2% enrichment)	↑ Nutritional quality, ↑ phytochemicals, antioxidant activity, ↑ color, appealing (2% best formulation)	[143]
Carrot pomace	Drying (60 °C, 5 h) → grinding (0.4 mm) → water-in-oil emulsion (addition of other ingredients + homogenization 5 min, 20 °C)	Butter (21% enrichment)	↑ Carbohydrates, proteins, vitamins, minerals, ↑ oxidative stability, ↓ microbial growth, ↓ spreadability, good acceptability	[144]
Red chili by- products	Freeze drying → grinding (0.5 mm) → carotenoid extraction: dispersion (1:100) in 60% acetone-40% petroleum ether (20 min stirring, protect from light) → centrifugation → solvent evaporation	Soybean oil (0.05–0.5 ppm enrichment)	↓ Oxidation, ↑ thermal stability	[129]
Bell pepper residue	Drying (60 °C, 16 h) → grinding (0.25 mm)	Sausage (50–270 mg GAE/kg meat)	↑ Ash, ↓ fat, ↑ lipid oxidative stability (180–2700 mg GAE/kg), natural additives (nitrite substitute)	[172]
Coffee husk ‘cascara’	Obtained commercially	Kombucha (10 g/L)	↑ Phenols, flavonoids, ↑ antioxidant, antibacterial activity, ↓ pH, balanced aroma and taste	[173]
Outer leaves of lettuce, onion peels, banana peels, whey protein, and brewery yeast	Freeze drying → grinding	Instant soups	↑ Antioxidant activity, ↑ phenols, flavonoids, hyperglycemia and hyperlipidemia, ↑ bone health, ↑ feces weight	[95]
Beet leaves	Washing → cutting → dispersion (1:50) in 80% ethanol (pH 6, 80 °C, 40 min) → centrifugation → solvent evaporation (40 °C) → resuspension in water	Vegetable smoothie (30% enrichment)	↑ TPC (+50%), ↑ antioxidant capacity, ↑ shelf life (+1 week), ↑ nutritional retention	[131]
Green tea leaves and branches, hops	MW hydrodiffusion and gravity/US/MW/ autohydrolysis (ethanol/water, 55–200 °C, 2–120 min)	Gluten-free hydrogel	Wide range of texture properties, ↑ thermal stability	[142]
Cowpea pod	Sun drying → grinding (0.25 mm) → water dispersion (1:15) → US treatment (20 kHz, 500 W, 10–20 min, 20–80% amplitude) → centrifugation → filtration (liquid) → freeze drying	Hydrogels	↑ TPC, ↑ antioxidant activity, ↑ beads size, ↓ compactness (in the presence of proteins)	[124]
Sesame, pomegranate, and grape seed	Grinding (1 mm) → sterilization (110 °C, 10 min, 1.5 atm)	Ice cream (2% enrichment)	↑ TPC, antioxidant activity (grape seed), ↑ viable probiotics	[146]
Peanut skin	Grinding (0.4 mm) → dispersion (1:10–30) in 95% ethanol → US treatment (10–60 min) → filtration → solvent evaporation (40 °C) → re-suspension in 95% ethanol	Dipped-bottom mushroom (0.005–0.05 mg/mL) and sacha inchi oil (0.01–0.05–1%)	↓ Oxidation, ↑ antioxidant activity	[127]
Rapeseed meal	Grinding (0.32 mm) → dispersion (1:4) in 75% ethanol (T °C room, 30 min, x4) → filtration → vacuum- concentration (50 °C) → freeze drying → phenols extraction: water dispersion (1:50, 20 min) → US treatment (20 kHz, 10–35% amplitude, 2–8 min) → centrifugation → filtration	Rapeseed oil (30% enrichment)	↓ Oxidation	[130]
Broccoli stalks, non-compliant carrots	Washing → disinfection → cutting → MW blanching (700 W, 2 min, x2) → cooling in ice → juice extraction	Beverage (82.5% broccoli, 17.5% carrots) (pasteurization, HPP, US treatment)	↑ Carotenoids, sulfur compounds, ↑ shelf life (HPP, US treatment)	[123]
Kimchi cabbage leaves and cortex	Steaming (120 °C, 11 min) → drying (80 °C, 48 h) → 0–100% methanol extraction → US treatment (0–30 min, 0–40% amplitude) → centrifugation (liquid) → freeze drying	Nanoparticles	↑ GLS protection (gastric phase), ↑ GLS release in intestine	[141]
Broccoli by-product	Obtained commercially	Beer (0.1% enrichment)	↑ Sulforaphane, ↑ alcohol content, ↓ bitterness, ↑ color, ↑ off-flavors, ↓ sensory properties	[151]
Non-compliant and broccoli stems	Blanching → freeze drying → grinding(powder) → dispersion (1:15) in 0–50% ethanol (40 °C, 1 h) → centrifugation (liquid extract)	Biscuit (10% flour, 30 mL extract/kg dough)	↑ Glucosinolates, TPC, carotenoids, color (flour), ↑ stickiness, ↓ workability (liquid extracts)	[121]
Brassica leaves	Washing → drying (60 °C, overnight)	Kombucha (7 g/L)	↓ TPC, antioxidant activity (than black tea), ↓ aroma, bitterness, ↑ color, appearance	[148]
Cauliflower stalks and leaves	Cutting → blanching (100 °C, 5 min) → freeze drying → grinding	Pizza (10–30% enrichment)	↑ Glucosinolates, carotenoids, phytosterols, good rheological properties (10%)	[16]
Broccoli stalks	Washing → cutting → brine	Fermented broccoli stalks	Source of phenols and GLS, ↓ broccoli flavor (fermentation), ↓ pH good sensory acceptability	[174]
Broccoli stem and leaves	Grinding (liquid nitrogen) → freeze drying	Salad dressing (1% enrichment)	↑ phenols bio-accessibility (modulated the presence of oil)	[145]
Fruit and vegetable waste	Quercetin extract powder obtained commercially	Bars (0.02–0.08% enrichment)	↑ Quercetin, TPC, ↑ antioxidant capacity, ↓ water activity, ↑ lightness, hardness, good sensory acceptability (0.06%)	[175]
Fruit and vegetable by-products	Washing → drying (60 °C, 5 h) → grinding → dispersion (1:3) in 96% ethanol (65 °C, 3 h) → filtration → concentration	Fish meal (300 ppm enrichment coffee sediment extract)	↑ Antioxidant activity, inhibits lipid oxidation	[126]

‘→’ followed by, ‘↓’ low, ‘↑’ high.

**Table 8:** Fish processing by-products with bioactive benefits and their food applications.

By-Products	Ingredient Preparation	Food Application	Properties	References
Skipjack tuna (*Katsuwonus pelamis*) by-products	Freeze drying → fat extraction (oil)	Pork sausage	↑ EPA (4.2%), DHA (22%), ↑ ABTS, DPPH, ↑ overall acceptance, ↑ antimicrobial properties	[154]
Mullet roe by-products	Three mild processes: pressure (PE) for fresh by-products, supercritical fluid extraction (SFE), and solvent extraction (SE) for freeze-dried by-products	Fish oil	↑ Antioxidant activity, DPPH, ABTS (SFE oil), ↑ oil yield (SE oil), ↑ EPA (20.7%), DHA (24.3%)	[156]
Fish by-products	Freeze drying → powder → microwave-assisted extraction (MAE)	Bioactive fish oil	↑ Oil yield (<19 min), no effect on DHA, linoleic, EPA, ↑ antimicrobial, antioxidant, anti-inflammatory activities	[157]
Catfish by-product	Fat extraction (US treatment)	Nanoemulsions (2 wt% oil)	↑ Antioxidant, antibacterial, anti-inflammatory properties, no toxicity against normal skin cells	[161]
Salmon (*Salmo salar*) backbones, heads, viscera	Freeze drying → powder → MAE: 14.6 min, 291.9 W, 80.1 g/L for backbones; 10.8 min, 50.0 W, 80.0 g/L for heads; and 14.3 min, 960.6 W, 99.5 g/L for viscera	Bioactive fish oil	↑ Oil recovery (69% heads, backbone; 92% viscera), ↑ cytotoxic, antioxidant, anti-inflammatory, and antimicrobial properties	[155]
Tuna (*Thunnus obesus*) and sea bass (*Dicentrarchus labrax*) by-product	4% Saline solution (5 min) → drying (50 °C) → grinding	Pasta (3% enrichment)	↑ DHA, EPA, cohesiveness (tuna), ↑ brightening (sea bass), ↓ hardness, fracturability, ↓ weight gain, swelling index	[176]
Northern shrimp (*Pandalus borealis*) by-product	Wet by-products → oil extraction (Soxhlet, hexane/acetone, 2:3)	Bioactive shrimp oil	↑ Phospholipids, n-3 PUFA and astaxanthin-esters, ↓ fat accumulation in 3T3-L1 cells, regulated adipogenesis and lipogenesis	[159]
Sardine (*Sardina pilchardus*) gill and viscera	Oil extraction (Soxhlet)	Chips (25 mL fish oil to 0.5 kg wheat)	↑ linoleic, linolenic, EPA, DHA, ↓ reducing triglycerides, LDL cholesterol, ↑ nutritional, antidiabetic, antihyperlipidemic, histoprotective effects	[158]
Nile perch (*Lates niloticus*) by-products	Oil extraction → centrifuging (recover of omega-3 FA)	Yoghurt (3.5 g oil)	Omega-3 daily intake was achieved through a single serving (150 g) of fortified yoghurt	[162]
Tuna, seabass, seabream, wild sardines by-products	Grinding → cooking (95 °C, 12 min → pressing → centrifuged (crude oil)	Crude oils	↓ Free fatty acid, peroxide, TOTOX, anisidine value (tuna, seabass, seabream) ↑ α-tocopherol (seabass, seabream), ↑ EPA, DHA (32.8% sardine oil)	[160]

‘→’ followed by, ‘↓’ low, ‘↑’ high.

**Table 9:** Dairy processing by-products with bioactive benefits and their food applications.

By-Products	Ingredient Preparation	Food Application	Properties	References
Sweet whey, sweet buttermilk, and skimmed milk	Pasteurization (72 °C, 15 s) → cooling → rennet addition → coagulation → draining → aging (4 °C, 24 h) → churning	Sweet whey fermented milk beverage, sweet buttermilk FMB, and skimmed milk FMB	↑ Antioxidant properties, ↓ glycemic index, glycemic load	[163]

‘→’ followed by, ‘↓’ low, ‘↑’ high.

## Data Availability

No new data were created or analyzed in this study. Data sharing is not applicable to this article.
